# Thyroid disease–attributed mortality burden and temporal trends among U.S. adults aged ≥65 years, 1999–2023: a nationwide analysis based on the CDC WONDER database

**DOI:** 10.3389/fpubh.2026.1869275

**Published:** 2026-07-30

**Authors:** Jiadi Li, Zide Chen

**Affiliations:** Department of Head and Neck Surgery, Sir Run Run Shaw Hospital, Zhejiang University School of Medicine, Hangzhou, Zhejiang, China

**Keywords:** attributed mortality burden, CDC WONDER database, nationwide analysis, temporal trends, thyroid disease

## Abstract

**Objective:**

To characterize temporal trends and sex-specific differences in mortality attributed to thyroid disease as the underlying cause among U.S. adults aged ≥65 years from 1999 to 2023 and to project mortality trajectories through 2030.

**Methods:**

We used the Centers for Disease Control and Prevention Wide-ranging Online Data for Epidemiologic Research (CDC WONDER) Multiple Cause of Death database to identify deaths among adults aged ≥65 years in which thyroid disease (International Classification of Diseases, 10th Revision codes E00–E07) was recorded as the underlying cause. Age-adjusted mortality rates (AAMRs) were calculated using the 2000 U.S. standard population. Temporal trends from 1999 to 2023 were evaluated using Joinpoint regression and log-linear models to estimate annual and average annual percent changes (APC/AAPC) and the estimated annual percentage change (EAPC). Autoregressive integrated moving average (ARIMA) and generalized additive models (GAM) were fitted to AAMR data from 1999 to 2020 to generate exploratory projections for 2021–2030.

**Results:**

A total of 41,660 thyroid disease–attributed deaths were identified. The overall AAMR decreased from 4.690 per 100,000 population in 1999 to 3.482 per 100,000 in 2023. The AAPC was −1.22%, and the EAPC was −1.43%, indicating a sustained decline over the study period. Women consistently had higher AAMRs than men, whereas men experienced a steeper decline in recent years. The ARIMA model projected a generally stable but gradually decreasing mortality trajectory through 2030. In contrast, the GAM captured nonlinear variation and suggested that the decline may attenuate or plateau during certain periods.

**Conclusion:**

Mortality attributed to thyroid disease among older adults in the United States declined over the past 25 years, although a measurable burden and persistent sex differences remain. These findings support continued surveillance and targeted thyroid assessment and management among high-risk older adults. The forecasting results should be interpreted as exploratory, model-based scenarios that may help inform long-term public health planning rather than as precise predictions of future mortality.

## Introduction

Thyroid disease is common among older adults and encompasses hypothyroidism, hyperthyroidism, thyroiditis, and other structural or functional disorders of the thyroid gland (1). Previous studies have shown that thyroid disorders may disrupt metabolic homeostasis and are associated with adverse outcomes, including cardiovascular disease, arrhythmias, osteoporosis, cognitive decline, and increased susceptibility to infection, all of which may contribute to a higher risk of death (2, 3). As the U.S. population continues to age, the number of adults aged ≥65 years is increasing, along with the cumulative burden of thyroid disease in this population. The potential contribution of thyroid disease to mortality among older adults therefore warrants closer attention.

Existing epidemiological studies of thyroid disease have primarily focused on prevalence, screening strategies, and cardiovascular risk (4–6). However, evidence regarding long-term temporal trends and differences across population subgroups in thyroid disease–attributed mortality remains limited (7, 8). Previous studies suggest that advances in diagnosis and treatment may have contributed to reductions in mortality associated with thyroid disease, although substantial disparities persist by sex, race and ethnicity, age, and geographic region (9, 10). Among older adults, multimorbidity, polypharmacy, and frailty may further complicate the recognition, treatment, and clinical consequences of thyroid disease. Moreover, the COVID-19 pandemic may have altered recent mortality patterns among older adults and disrupted preexisting trends in thyroid disease–attributed mortality (11).

From a methodological perspective, previous studies relied on a single approach to trend analysis and have focused primarily on overall or short-term changes. Relatively few studies have characterized long-term nonlinear patterns or explored future mortality trajectories (12–14). For public health planning and resource allocation, nationally representative data are needed not only to describe historical trends but also to generate cautious, model-based projections of possible future changes. This is particularly relevant in the United States, where population aging and regional health disparities may contribute to variation in thyroid disease–attributed mortality among older adults.

Accordingly, we used the Centers for Disease Control and Prevention Wide-ranging Online Data for Epidemiologic Research (CDC WONDER) Multiple Cause of Death database to identify deaths from 1999 to 2023 in which thyroid disease (ICD-10 codes E00–E07) was recorded as the underlying cause among adults aged ≥65 years (15). We examined long-term changes in crude and age-adjusted mortality rates (AAMRs) and conducted stratified analyses by sex, race and ethnicity, age group, census region, state, and urban–rural category. We also assessed the distribution of common contributing causes of death. Temporal trends were evaluated using Joinpoint regression to estimate annual percentage changes and average annual percentage changes, together with log-linear models to estimate the estimated annual percentage change. In addition, autoregressive integrated moving average and generalized additive models were fitted to AAMR data from 1999 to 2020 to generate exploratory projections for 2021–2030, providing complementary linear and nonlinear scenarios. Through these analyses, we aimed to characterize the burden, temporal patterns, and population disparities in thyroid disease–attributed mortality among older adults in the United States and to provide evidence for targeted clinical management, continued surveillance, and public health planning.

## Materials and methods

### Study design and data source

This nationwide, population-based study used a time-series design with forecasting analyses. Data were obtained from the Multiple Cause of Death files in the CDC WONDER system for 1999–2023. Maintained by the National Center for Health Statistics within the U.S. Centers for Disease Control and Prevention, this database contains information derived from death certificates of U.S. residents, including the underlying cause of death, contributing causes, and demographic and geographic characteristics. Using the CDC WONDER online interface (https://wonder.cdc.gov/), we extracted death records from January 1, 1999, and December 31, 2023, among individuals aged ≥65 years for whom thyroid disease was listed as the underlying cause of death. These data were used to assess long-term temporal trends and to develop forecasting models for thyroid disease–attributed mortality. Because CDC WONDER provides publicly available, de-identified, aggregate data, institutional review board approval and informed consent were not required.

### Definition of thyroid disease–related deaths and classification of contributing causes

#### Thyroid disease–attributed deaths

According to the International Classification of Diseases, 10th Revision (ICD-10), we defined thyroid diseases as codes E00–E07, encompassing hypothyroidism, hyperthyroidism, and other structural and functional thyroid disorders (16). Thyroid cancer was excluded because malignant neoplasm of the thyroid gland is classified separately as ICD-10 code C73. We identified deaths among adults aged ≥65 years in which thyroid disease was recorded as the underlying cause. Deaths were included regardless of any additional contributing causes recorded on the death certificate. Accordingly, the study outcome was defined as mortality for which thyroid disease was recorded as the underlying cause of death, hereafter referred to as thyroid disease–attributed mortality.

#### Classification of contributing causes

To characterize other conditions recorded on the death certificates, we retrieved all additional multiple or contributing causes and grouped them into major ICD-10 categories (17, 18): Neoplasms (C00–D48); Diseases of the circulatory system (I00–I99); Endocrine, nutritional and metabolic diseases (E10–E88); Mental and behavioural disorders (F01–F99); Diseases of the nervous system (G00–G98); Diseases of the respiratory system (J00–J98); Diseases of the digestive system (K00–K92). Annual proportions of these categories were calculated to describe the distribution and temporal changes in co-occurring causes of death among older adults for whom thyroid disease was recorded as the underlying cause.

### Study population and stratification variables

#### Inclusion criteria

Deaths were included if they met all of the following criteria: (1) the death occurred between 1999 and 2023; (2) age at death was ≥65 years; (3) thyroid disease, defined by ICD-10 codes E00–E07, was recorded as the underlying cause of death, and (4) the decedent’s usual residence was within one of the 50 U.S. states.

#### Demographic stratification

Stratified analyses were conducted according to sex, race/ethnicity, and age group using variables available in CDC WONDER. The study was restricted to adults aged ≥65 years because this threshold is commonly used to define older adults in epidemiological and public health research and has been applied in prior CDC WONDER-based mortality analyses, thereby supporting methodological consistency and comparability. Sex was categorized as male or female. Race and ethnicity were classified as non-Hispanic White, non-Hispanic Black, non-Hispanic Other, or Hispanic. Age at death was grouped as 65–74 years, 75–84 years, or ≥85 years to assess heterogeneity within the older population.

#### Geographic and urban–rural stratification

Following U.S. Census Bureau and CDC WONDER classifications, the United States was divided into four regions: Northeast, Midwest, South, and West; State-specific thyroid disease–related mortality rates were calculated for each of the 50 states. County urbanization level was classified using the 2013 National Center for Health Statistics Urban–Rural Classification Scheme for Counties, which comprises six categories: large central metropolitan, large fringe metropolitan, medium metropolitan, small metropolitan, micropolitan, and noncore counties. For the primary analysis, these categories were combined into three groups: large metropolitan areas, comprising large central and large fringe metropolitan counties; medium/small metropolitan areas, comprising medium and small metropolitan counties; and rural areas, comprising micropolitan and noncore counties. In secondary binary comparisons, the four metropolitan categories were combined as urban, whereas micropolitan and noncore counties were combined as rural.

### Mortality measures

#### Crude mortality rate (CMR)

Annual crude mortality rates (CMRs) for thyroid disease–attributed deaths were calculated as (19–21):


$$\mathrm{CMR}=\frac{\begin{array}{l} \text{Number}\;\;\text{of thyroid disease}\;\\ -\text{related deaths in}\;a\;\text{given year} \end{array}}{\text{Corresponding}\;U.S.\text{population in that year}}\times 100,000$$

Annual population estimates were obtained from the population denominators embedded in CDC WONDER.

#### Age-adjusted mortality rate (AAMR)

To allow comparison over time while accounting for changes in age structure, we calculated age-adjusted mortality rates (AAMRs) using the 2000 U.S. standard population via direct standardization (22, 23). AAMRs were expressed per 100,000 persons and reported together with 95% confidence intervals (CIs).

#### Percent change (PC)

To quantify relative changes in AAMR between selected time points, we computed PC as (24):


$$\mathrm{PC}=\frac{\text{AAMRend}-\text{AAMRstart}}{\text{AAMRstart}}\times 100\%$$

### Trend analysis

#### Joinpoint regression and annual percent change (APC)

To describe long-term temporal trends in AAMR and detect potential inflection points, we applied the Joinpoint Regression Program (National Cancer Institute, USA) for the period 1999–2023 (25, 26). Log-linear models with zero or more joinpoints were fitted, and the best-fitting model was selected using Monte Carlo permutation tests. For each identified time segment, the annual percent change (APC) and its 95% CI were estimated to characterize segment-specific trends. The average annual percent change (AAPC) was calculated as a weighted average of segment-specific APCs and used to summarize the overall trend over the full study period or a prespecified interval. Trends were considered statistically significant only when the corresponding 95% CI did not include zero. Specifically, if the 95% CI for APC or AAPC was entirely above zero, the trend was considered significantly increasing; if entirely below zero, significantly decreasing; and if it included zero, the trend was considered not statistically significant.

#### Estimated annual percentage change (EAPC)

To provide an overall trend measure independent of segmented models, we additionally calculated the EAPC based on a log-linear regression of AAMR on calendar year (27, 28):


ln(AAMR)=α+β×Year+ε

Where Year denotes calendar year (1999–2023) and β is the slope parameter.

The EAPC was then derived as:

EAPC = 100 × ( $e^{\beta }-1$ )

with 95% CIs derived from the standard error of β. A 95% CI entirely above or below 0 indicates a statistically significant increasing or decreasing trend, respectively.

### Forecasting models

To reduce the potential influence of the COVID-19 pandemic on model stability, forecasting models were trained using annual AAMR data from 1999 to 2020, and predictions were generated for 2021–2030.

#### Autoregressive integrated moving average (ARIMA) model

We used the auto.arima() function from the R forecast package (https://github.com/robjhyndman/forecast) to fit ARIMA models to the 1999–2020 AAMR series (29, 30). The “auto.arima()” function automatically searches through combinations of orders (p, d, q); The optimal model was selected based on the lowest Akaike information criterion (AIC) and Bayesian information criterion (BIC) values; Model residuals were assessed using the Ljung–Box test; a *p*-value >0.05 indicated no significant autocorrelation and adequate model fit; The selected ARIMA model was then used to forecast AAMRs for 2021–2030, with point predictions and 95% prediction intervals.

#### Generalized additive model (GAM)

To capture potential nonlinear temporal patterns, we fitted a generalized additive model (GAM) using the mgcv package in R (31–33):


AAMR~s(Year,k=10)

Where s (Year) represents a smooth spline term for calendar year, with an upper limit of 10 basis functions to reduce overfitting. The smoothing parameter was estimated using restricted maximum likelihood (REML).

We reported the effective degrees of freedom (edf), F-statistic, and *p*-value for the smooth term, as well as the deviance explained and adjusted R^2^ as measures of model fit. The fitted GAM was then used to predict AAMRs for 2021–2030, and 95% CIs for the predictions were obtained. By comparing the trajectories and uncertainty intervals from ARIMA and GAM, we provided complementary linear and nonlinear perspectives on potential future trends in thyroid disease–related mortality among older adults.

### Statistical analysis

Apart from Joinpoint regression, which was conducted using the dedicated Joinpoint software, all statistical analyses and graphical displays were performed using R (version 4.2.3). Continuous variables were summarized as annual time-series values. APC, AAPC, and EAPC and their 95% CIs were computed as described above. All statistical tests were two-sided, and a *p*-value < 0.05 was considered statistically significant. Where applicable, statistically significant differences were indicated by an asterisk (*) in figures and tables.

## Results

### Overall trends

From 1999 through 2023, a total of 41,660 deaths among U.S. adults aged ≥65 years were attributed to thyroid diseases (ICD-10: E00–E07) as the underlying cause of death (Table 1). Over this 25-year period, the overall AAMR showed a declining pattern, decreasing from 4.690 per 100,000 (95% CI 4.460–4.920) in 1999 to 3.482 per 100,000 (95% CI 3.324–3.641) in 2023. Taking 1999 as the reference, the long-term PC in AAMR by 2023 was approximately −24.95%, indicating a substantial reduction in thyroid disease–related mortality burden among older adults. Further analysis revealed that the mortality decline occurred primarily before 2004 (decreasing from a peak of 4.83 per 100,000 to 4.22 per 100,000), followed by a period of relative stability from 2004 to 2020 (AAMR fluctuating between 3.3–3.9 per 100,000), with a slight upward trend observed in the most recent three years (2021–2023, AAMR ranging from 3.48–3.67 per 100,000).

**Table 1:** Age-adjusted mortality rates (AAMR) from thyroid diseases (ICD-10: E00–E07) among U.S. adults aged ≥65 years, 1999–2023.

Year	Deaths	Population	Crude rate (95%CI)	SE	AAMR	SE	Total deaths (%)
1999	1,597	34,797,841	4.59 (4.36 ~ 4.81)	0.11	4.69 (4.46 ~ 4.92)	0.12	4.43%
2000	1,544	34,991,753	4.41 (4.19 ~ 4.63)	0.11	4.44 (4.22 ~ 4.66)	0.11	4.29%
2001	1703	35,290,291	4.83 (4.60 ~ 5.05)	0.12	4.82 (4.59 ~ 5.05)	0.12	4.73%
2002	1715	35,522,207	4.83 (4.60 ~ 5.06)	0.12	4.77 (4.55 ~ 5.00)	0.12	4.76%
2003	1707	35,863,529	4.76 (4.53 ~ 4.99)	0.12	4.70 (4.47 ~ 4.92)	0.11	4.74%
2004	1,529	36,203,319	4.22 (4.01 ~ 4.44)	0.11	4.18 (3.97 ~ 4.38)	0.11	4.25%
2005	1700	36,649,798	4.64 (4.42 ~ 4.86)	0.11	4.53 (4.31 ~ 4.75)	0.11	4.72%
2006	1,584	37,164,107	4.26 (4.05 ~ 4.47)	0.11	4.12 (3.92 ~ 4.32)	0.10	4.40%
2007	1,534	37,825,711	4.06 (3.85 ~ 4.26)	0.10	3.87 (3.68 ~ 4.06)	0.10	4.26%
2008	1,534	38,777,621	3.96 (3.76 ~ 4.15)	0.10	3.80 (3.61 ~ 3.99)	0.10	4.26%
2009	1,441	39,623,175	3.64 (3.45 ~ 3.82)	0.10	3.51 (3.33 ~ 3.70)	0.09	4.00%
2010	1,567	40,267,984	3.89 (3.70 ~ 4.08)	0.10	3.73 (3.54 ~ 3.91)	0.09	4.35%
2011	1,560	41,394,141	3.77 (3.58 ~ 3.96)	0.10	3.55 (3.38 ~ 3.73)	0.09	4.33%
2012	1,615	43,145,356	3.74 (3.56 ~ 3.93)	0.09	3.62 (3.44 ~ 3.79)	0.09	4.48%
2013	1,571	44,704,074	3.51 (3.34 ~ 3.69)	0.09	3.41 (3.24 ~ 3.58)	0.09	4.36%
2014	1702	46,243,211	3.68 (3.51 ~ 3.86)	0.09	3.63 (3.45 ~ 3.80)	0.09	4.73%
2015	1,628	47,760,852	3.41 (3.24 ~ 3.57)	0.08	3.38 (3.22 ~ 3.55)	0.08	4.52%
2016	1,593	49,244,195	3.23 (3.08 ~ 3.39)	0.08	3.26 (3.10 ~ 3.42)	0.08	4.42%
2017	1,698	50,858,679	3.34 (3.18 ~ 3.50)	0.08	3.40 (3.24 ~ 3.56)	0.08	4.71%
2018	1785	52,431,193	3.40 (3.25 ~ 3.56)	0.08	3.48 (3.32 ~ 3.64)	0.08	4.96%
2019	1816	54,058,263	3.36 (3.20 ~ 3.51)	0.08	3.44 (3.28 ~ 3.60)	0.08	5.04%
2020	1891	55,659,365	3.40 (3.24 ~ 3.55)	0.08	3.52 (3.36 ~ 3.68)	0.08	5.25%
2021	1828	55,847,953	3.27 (3.12 ~ 3.42)	0.08	3.63 (3.46 ~ 3.79)	0.09	32.38%
2022	1956	57,794,852	3.38 (3.23 ~ 3.53)	0.08	3.67 (3.50 ~ 3.83)	0.08	34.64%
2023	1862	59,248,361	3.14 (3.00 ~ 3.29)	0.07	3.48 (3.32 ~ 3.64)	0.08	32.98%

AAMR, age-adjusted mortality rate per 100,000 person-years; 95% CI, 95% confidence interval; SE, standard error; ICD-10, international classification of diseases, 10th revision.

Stratified analysis by demographic and geographic characteristics (Table 2) revealed heterogeneous trends in thyroid disease–related mortality. By age group, the 75–84 years and ≥85 years subgroups showed significant declines in AAMR (AAPC: −1.53 and −1.63, respectively; *p* < 0.05), while the 65–74 years group demonstrated a modest increasing trend (AAPC: 0.85; *p* = 0.033). Joinpoint regression indicated a statistically significant downward trend in overall AAMR during 1999–2023, with an AAPC of −1.22 (95% CI − 1.64 to −0.84; *p* < 0.05). The log-linear model yielded an estimated annual percentage change (EAPC) of −1.43 (95% CI − 1.82 to −1.04; *p* < 0.05), consistent with the AAPC and confirming a modest but persistent decline in thyroid disease–related mortality among older adults over the study period. By geographic region, all census regions showed declining trends, with the Midwest demonstrating the steepest decrease (AAPC: −1.73), followed by the Northeast (−1.25), West (−0.87), and South (−0.79). Racial/ethnic analysis indicated significant declines among Non-Hispanic White (−0.97) and Non-Hispanic Black (−0.97) populations, while Hispanic (−0.58) and Non-Hispanic Other (0.58) populations showed non-significant trends. Sex-stratified analysis revealed comparable declines between females (AAPC: −1.11) and males (−1.04). By urbanization level, metropolitan areas exhibited the most pronounced decline (AAPC: −2.16), followed by medium/small metropolitan areas (−1.19) and rural counties (−1.01).

**Table 2:** Age-adjusted mortality rates (AAMR) and trends in thyroid disease–related deaths among U.S. adults aged ≥65 years by demographic and geographic characteristics, 1999–2023.

Variables	Deaths (1999)	Deaths (2023)	Change (%)	AAMR_1999_ (95% CI)	AAMR_2023_ (95% CI)	AAPC (95% CI)	*P*
Age group (years)							
65–74	177	337	8.33	0.96 (0.82 ~ 1.1)	1.04 (0.92 ~ 1.15)	0.85 (0.07 ~ 1.77)	0.033
75–84	488	449	−31.58	3.99 (3.64 ~ 4.35)	2.73 (2.48 ~ 2.98)	−1.53 (−2.04 ~ −1.06)	<0.001
85+	932	1,105	−26.02	22.44 (21 ~ 23.88)	16.6 (15.62 ~ 17.57)	−1.63 (−2.17 ~ −1.19)	<0.001
Census region							
Northeast	360	398	−19.71	4.82 (4.32 ~ 5.32)	3.87 (3.49 ~ 4.25)	−1.25 (−1.87 ~ −0.55)	0.002
Midwest	428	401	−30.43	5.06 (4.58 ~ 5.53)	3.52 (3.18 ~ 3.87)	−1.73 (−2.05 ~ −1.32)	<0.001
South	583	781	−19.96	4.96 (4.56 ~ 5.37)	3.97 (3.69 ~ 4.24)	−0.79 (−1.12 ~ −0.45)	<0.001
West	226	311	−23.41	3.46 (3.01 ~ 3.91)	2.65 (2.35 ~ 2.95)	−0.87 (−1.48 ~ −0.29)	0.004
Race							
Hispanic	48	136	−15.64	3.58 (2.63 ~ 4.76)	3.02 (2.51 ~ 3.54)	−0.58 (−1.42 ~ 0.28)	0.201
NH black	129	197	−14.89	4.97 (4.11 ~ 5.83)	4.23 (3.63 ~ 4.83)	−0.97 (−1.55 ~ −0.33)	0.002
NH other	18	59	NA	NA (1.39 ~ 3.82)	2.05 (1.56 ~ 2.65)	0.58 (−0.76 ~ 2.13)	0.323
NH white	1,400	1,490	−22.93	4.71 (4.47 ~ 4.96)	3.63 (3.44 ~ 3.81)	−0.97 (−1.33 ~ −0.47)	<0.001
Sex							
Female	1,265	1,439	−19.32	5.59 (5.28 ~ 5.9)	4.51 (4.27 ~ 4.74)	−1.11 (−1.52 ~ −0.74)	<0.001
Male	332	452	−27.66	2.82 (2.51 ~ 3.13)	2.04 (1.85 ~ 2.23)	−1.04 (−1.65 ~ −0.39)	<0.001
Urbanization							
Med-small	504	633	−21.95	4.83 (4.41 ~ 5.26)	3.77 (3.47 ~ 4.06)	−1.19 (−1.57 ~ −0.88)	<0.001
Metropolitan	806	906	−30.59	4.74 (4.41 ~ 5.07)	3.29 (3.07 ~ 3.5)	−2.16 (−2.67 ~ −1.63)	<0.001
Rural	287	352	−6.38	4.23 (3.74 ~ 4.72)	3.96 (3.55 ~ 4.38)	−1.01 (−1.65 ~ −0.32)	0.006

AAMR, age-adjusted mortality rate; AAPC, average annual percent change; CI, confidence interval; EAPC, estimated annual percent change; ICD-10, international classification of diseases, 10th revision; NH, non-hispanic.

### Sex-stratified analysis

**Figure 1:**
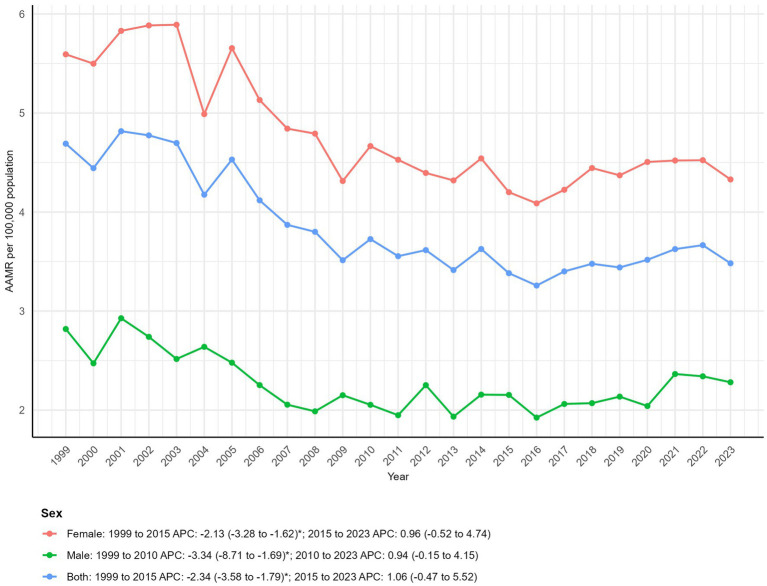
Age-standardized mortality rate of thyroid disease per 100,000 population, overall and stratified by sex, 1999–2023.

Sex-stratified analyses revealed substantial differences in absolute mortality burden and temporal trends between men and women (Table 2; Figure 1; Supplementary Table S1). Among the 41,660 total deaths from 1999–2023, 9,236 deaths (22.17%) occurred in men and 32,424 deaths (77.83%) in women, reflecting the predominance of thyroid disease mortality among women in older age groups.

Men experienced lower absolute mortality rates than women throughout the entire study period. Among men, the AAMR decreased from 2.82 per 100,000 (95% CI 2.51–3.13) in 1999 to 2.04 per 100,000 (95% CI 1.85–2.23) in 2023, representing a percent change of approximately −27.66%. Women, who consistently experienced higher absolute mortality rates, showed decline from 5.59 per 100,000 (95% CI 5.28–5.90) in 1999 to 4.51 per 100,000 (95% CI 4.27–4.74) in 2023, with a percent change of approximately −19.32%. The female-to-male mortality ratio remained relatively stable throughout the study period at approximately 2.0–2.5, suggesting persistent sex-based differences in thyroid disease mortality burden that are maintained despite recent trend reversals.

Both sexes demonstrated significant declines through 2015, followed by divergent patterns. Among men, Joinpoint analysis showed an overall AAPC of −1.04 (95% CI − 1.65 to −0.39; *p* < 0.05), with steeper declines during 1999–2010 (APC: −3.34; 95% CI − 8.71 to −1.69), followed by modest increases from 2010–2023 (APC: 0.94; 95% CI − 0.15 to 4.15). The EAPC of −0.96 (95% CI − 1.53 to −0.39; *p* < 0.05) confirmed the overall downward trend and its robustness. Women showed significant decline from 1999–2015 (APC: −2.13; 95% CI − 3.28 to −1.62), followed by stabilization with modest increases during 2015–2023 (APC: 0.96; 95% CI − 0.52 to 4.74). The corresponding AAPC of −1.11 (95% CI − 1.52 to −0.74; *p* < 0.05) and EAPC of −1.30 (95% CI − 1.69 to −0.92; *p* < 0.05) supported a sustained decreasing trend overall.

#### Age-stratified trends

**Figure 2:**
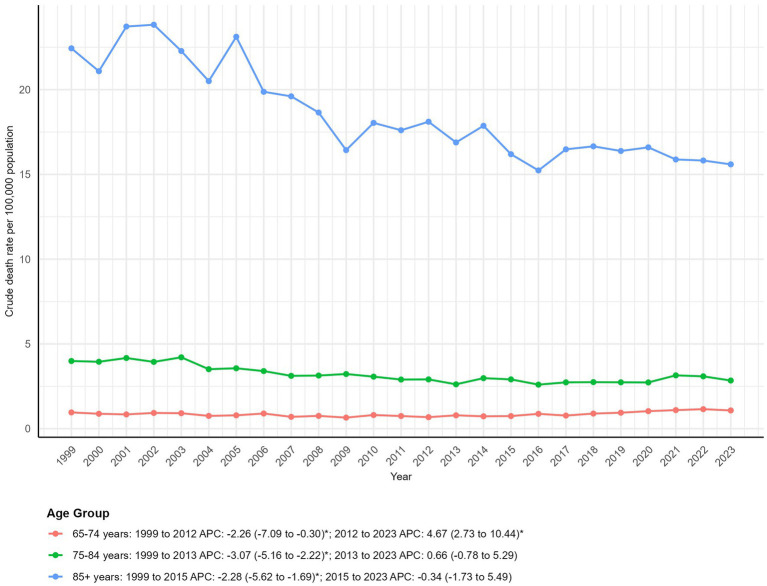
Crude mortality rate of thyroid disease per 100,000 population, stratified by age group, 1999–2023.

Age-stratified analysis revealed distinct mortality patterns across age groups (Figure 2; Supplementary Table S1). The 85 + years age group exhibited the highest absolute mortality rates throughout the study period, with AAMR ranging from 22.44 per 100,000 (1999) to 15.59 per 100,000 (2023). This group demonstrated a significant decline during 1999–2015 (APC: −2.28; 95% CI − 5.62 to −1.69), followed by stabilization from 2015–2023 (APC: −0.34; 95% CI − 1.73 to 0.49). The 75–84 years cohort showed a comparable downward trend during 1999–2013 (APC: −3.07; 95% CI − 5.16 to −2.22), with mortality rates stabilizing thereafter (2013–2023 APC: 0.66; 95% CI − 0.78 to 5.29). In contrast, the 65–74 years group, which had the lowest mortality rates, demonstrated an initial decline from 1999–2012 (APC: −2.26; 95% CI − 7.09 to −0.30), followed by a remarkable reversal with statistically significant increases during 2012–2023 (APC: 4.67; 95% CI 2.73 to 10.44). This age-group-specific upward trend in the youngest older adults warrants further investigation into recent epidemiological changes.

To evaluate the potential influence of the COVID-19 pandemic on mortality trends, we conducted a sensitivity analysis restricted to the pre-pandemic period from 1999 to 2019 (Supplementary Table S2). In this analysis, the overall AAMR for deaths with thyroid disease recorded as the underlying cause decreased from 4.69 per 100,000 in 1999 to 3.44 per 100,000 in 2019, corresponding to a percent change of −26.65%. The AAPC was −1.52% (95% CI: −1.85 to −1.10), and the EAPC was −1.95% (95% CI: −2.34 to −1.55). Declining trends were also observed among both women and men, with AAPCs of −1.42% (95% CI: −2.10 to −0.95) and −1.72% (95% CI: −2.41 to −0.92), respectively. Similar downward patterns were observed across most age groups, racial/ethnic groups, census regions, and urbanization categories. These findings were generally consistent with the main analysis and suggest that the long-term decline in thyroid disease-attributed mortality was not solely driven by pandemic-era mortality fluctuations.

#### Geographic and regional variation

**Figure 3:**
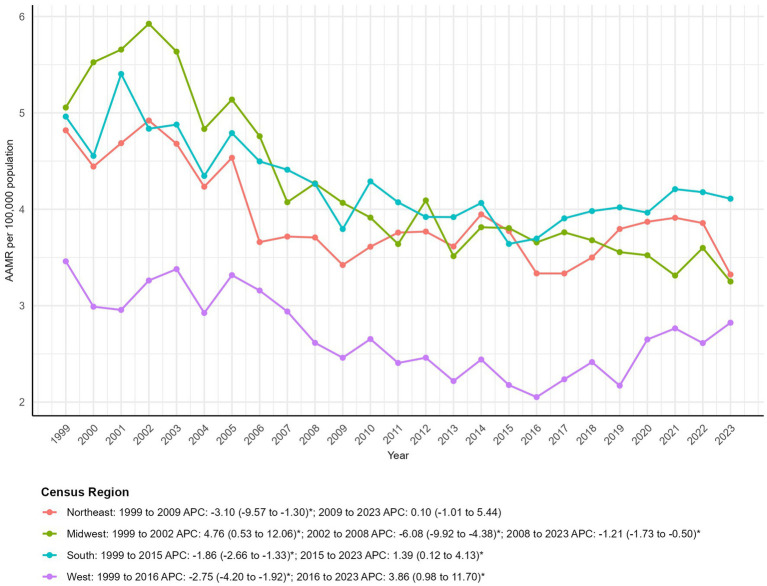
Age-standardized mortality rate of thyroid disease per 100,000 population, stratified by census region, 1999–2023.

Regional analysis identified substantial geographic heterogeneity in thyroid disease mortality trends (Figure 3; Supplementary Table S1). The Midwest region displayed the most dramatic early increase, rising from 5.06 per 100,000 (1999) to 5.92 per 100,000 (2003) with APC of 4.76 (95% CI 0.53 to 12.06), followed by substantial declines from 2002–2008 (APC: −6.08; 95% CI − 9.92 to −4.38). The Northeast region showed consistent decline from 1999–2009 (APC: −3.10; 95% CI − 9.57 to −1.30), with mortality stabilizing at approximately 3.3–3.9 per 100,000 from 2009 onward. The South region demonstrated a more prolonged decline through 2015 (APC: −1.86; 95% CI − 2.66 to −1.33), followed by recent increases (2015–2023 APC: 1.39; 95% CI 0.12 to 4.13). The West region exhibited similar patterns, declining steadily until 2016 (APC: −2.75; 95% CI − 4.20 to −1.92), with subsequent increases during 2016–2023 (APC: 3.86; 95% CI 0.98 to 11.70). These regional divergences suggest that state-level and local healthcare factors may differentially influence thyroid disease mortality outcomes.

#### Racial and ethnic disparities

**Figure 4:**
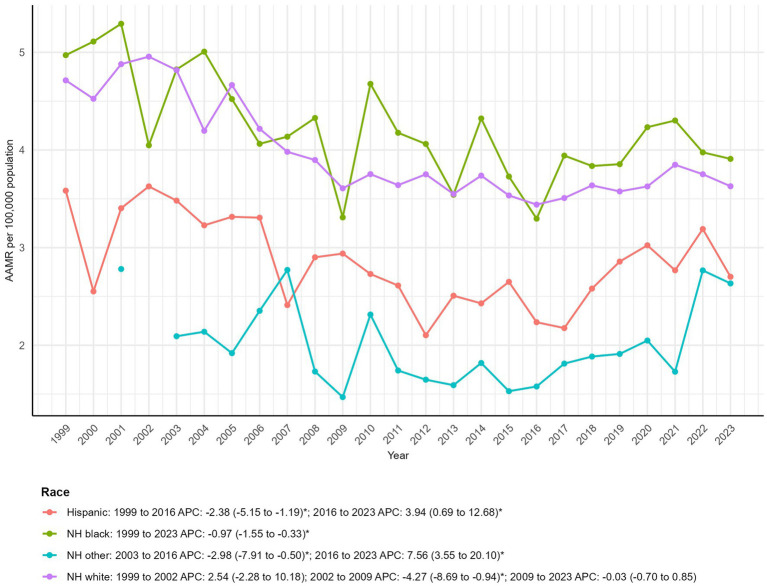
Age-standardized mortality rate of thyroid disease per 100,000 population, stratified by race and ethnicity, 1999–2023.

Racial/ethnic stratification revealed important disparities in mortality trends (Figure 4; Supplementary Table S1). Non-Hispanic Black populations maintained the most consistent downward trend throughout the entire 1999–2023 period (APC: −0.97; 95% CI − 1.55 to −0.33), declining from 4.97 per 100,000 (1999) to 3.91 per 100,000 (2023). Non-Hispanic White populations, the largest subgroup, showed complex temporal patterns with initial increases from 1999–2002 (APC: 2.54; 95% CI − 2.28 to 10.18), sharp declines from 2002–2009 (APC: −4.27; 95% CI − 8.69 to −0.94), and relative stabilization from 2009–2023 (APC: −0.03; 95% CI − 0.70 to 0.85). Hispanic populations demonstrated a significant decline from 1999–2016 (APC: −2.38; 95% CI − 5.15 to −1.19), with a concerning reversal from 2016–2023 (APC: 3.94; 95% CI 0.69 to 12.68). The Non-Hispanic Other category showed limited early data but demonstrated substantial increases during 2016–2023 (APC: 7.56; 95% CI 3.55 to 20.10), albeit with smaller absolute numbers. These patterns suggest that race/ethnicity-specific factors, potentially including healthcare access, health literacy, or disease management practices, may contribute to differential mortality outcomes.

#### Urbanization-based differences

**Figure 5:**
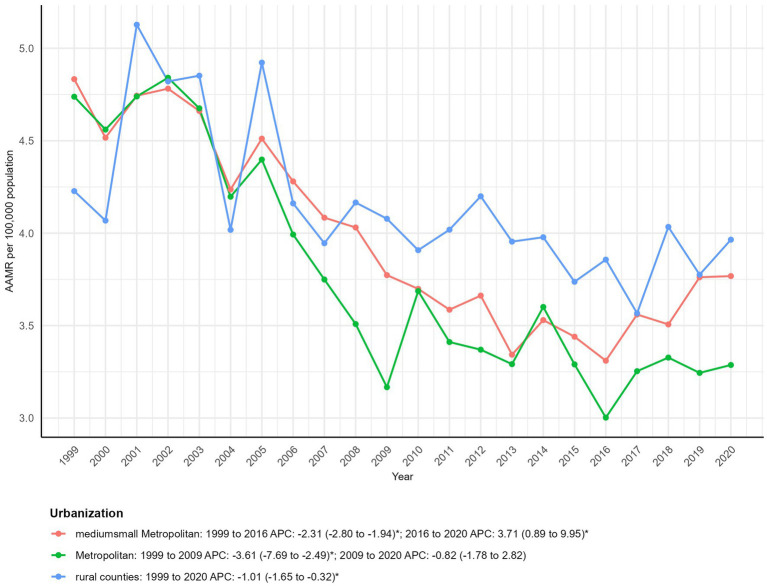
Age-standardized mortality rate of thyroid disease per 100,000 population, stratified by urbanization level, 1999–2023.

Urbanization status revealed important geographic-economic disparities in mortality patterns (Figure 5; Supplementary Table S1). Metropolitan areas exhibited the steepest decline during 1999–2009 (APC: −3.61; 95% CI − 7.69 to −2.49), with subsequent stabilization from 2009–2020 (APC: −0.82; 95% CI − 1.78 to 0.82). Medium/small metropolitan areas demonstrated more modest initial decline from 1999–2016 (APC: −2.31; 95% CI − 2.80 to −1.94), followed by more pronounced increases from 2016–2020 (APC: 3.71; 95% CI 0.89 to 9.95). Rural counties, which consistently exhibited the highest mortality rates throughout the study period (ranging from 4.23 to 3.96 per 100,000), showed steady but less dramatic declines from 1999–2020 (APC: −1.01; 95% CI − 1.65 to −0.32). The persistent higher mortality rates in rural areas suggest potential disparities in access to thyroid disease screening, diagnosis, and management in less urbanized regions.

#### State-level variation

**Figure 6:**
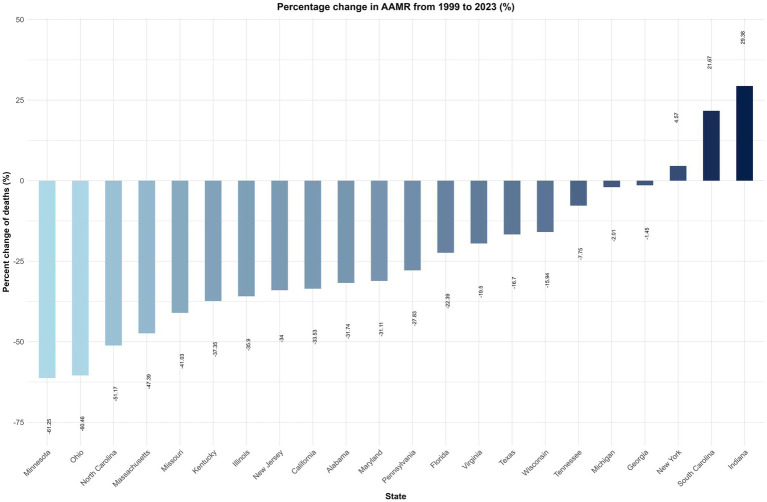
Percentage change in state-level age-standardized mortality rate of thyroid disease per 100,000 population, 1999–2023.

State-level analysis demonstrated marked heterogeneity in mortality trajectories, with percentage changes in AAMR from 1999 to 2023 ranging from −61.25% (Minnesota) to +29.38% (Indiana) (Figure 6; Supplementary Table S1). States with the most substantial reductions included Minnesota (−61.25%), Ohio (−60.46%), North Carolina (−51.17%), Massachusetts (−47.39%), Missouri (−41.03%), and New Jersey (−34.00%). These states, distributed across multiple regions and urbanization levels, may represent best-practice models for thyroid disease mortality reduction. Conversely, Indiana (29.38%), South Carolina (21.67%), and Connecticut (14.54%) experienced notable increases over the 25-year period. Several states with historically sparse data (Alaska, Hawaii, North Dakota, Rhode Island, South Dakota, Utah, Vermont, and Wyoming) had insufficient deaths for trend analysis. The substantial state-level variation (ranging across a 90-percentage-point spectrum) suggests that state-specific factors—including healthcare infrastructure, screening practices, thyroid disease epidemiology, and/or mortality coding practices—significantly influence population-level outcomes.

#### Forecasting model results

**Figure 7:**
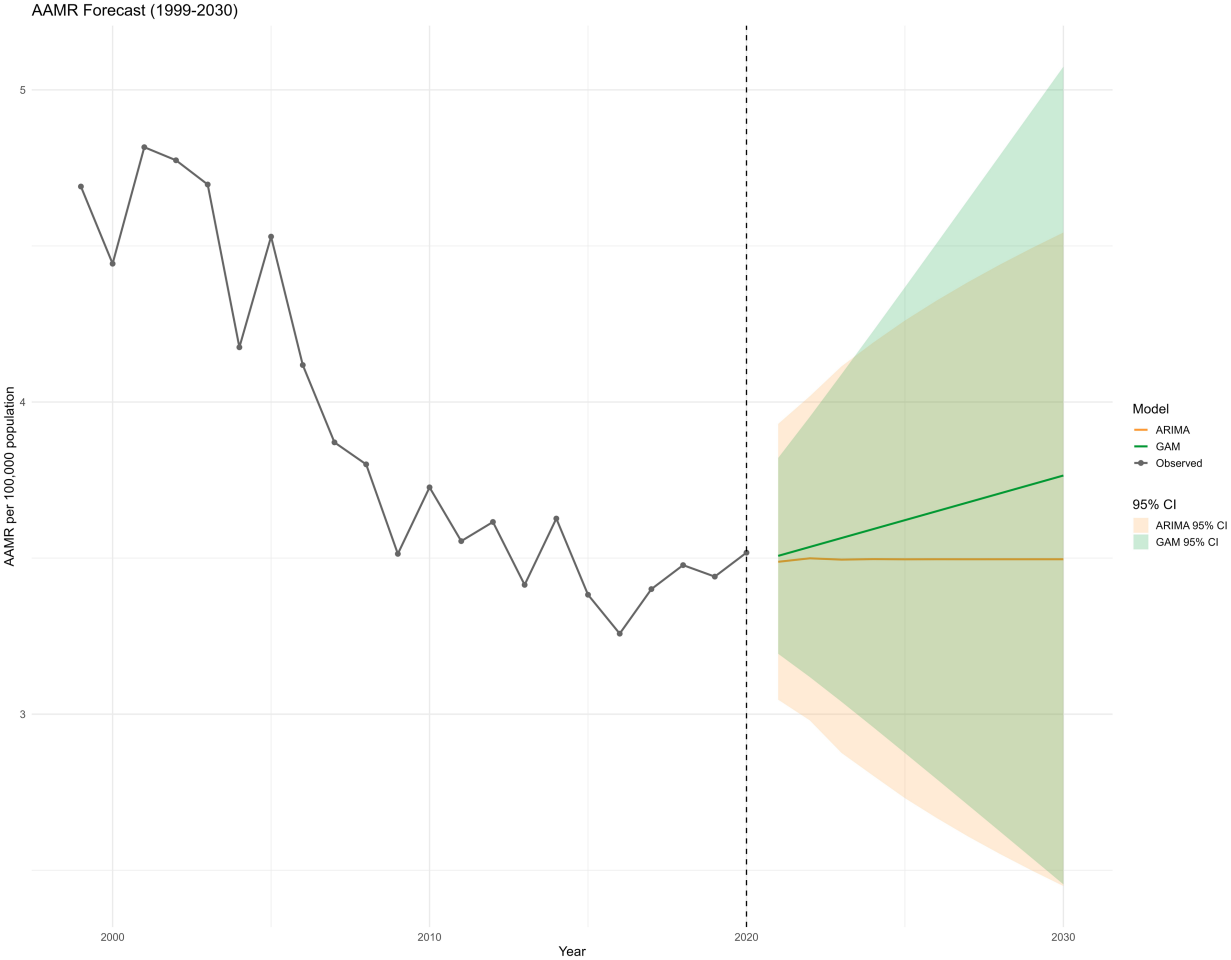
Observed and forecasted trends in the overall age-standardized mortality rate of thyroid disease from 1999 to 2030. Observed estimates were available for 1999–2020, and forecasts for 2021–2030 were generated using autoregressive integrated moving average (ARIMA) and generalized additive models (GAMs), with shaded areas representing 95% confidence intervals.

To contextualize the observed trends and inform future public health planning, we developed two complementary time-series forecasting models based on AAMR data from 1999 to 2020: an Autoregressive Integrated Moving Average (ARIMA) model and a Generalized Additive Model (GAM). These approaches offer different perspectives on potential future trajectories of thyroid disease–related mortality among older adults through 2030 (Figure 7).

#### ARIMA model

The auto.arima() procedure selected ARIMA(1,1,0) as the optimal model based on iterative evaluation of Akaike Information Criterion (AIC) and Bayesian Information Criterion (BIC). The selected model yielded a low AIC (0.15) and BIC (2.24), indicating good model parsimony and efficiency. The Ljung–Box test for residual autocorrelation produced a *p*-value >0.05, suggesting no significant residual autocorrelation and confirming adequate model fit. The root mean squared error (RMSE) was 0.215, reflecting relatively small fitting errors and good retrospective predictive accuracy.

Based on this ARIMA(1,1,0) specification, forecasts of AAMR for 2021–2030 displayed an approximately linear, gently upward trajectory without prominent inflection points (Figure 7, orange line). The 95% confidence interval for ARIMA predictions remained relatively narrow throughout the forecast period, suggesting stable and precise model estimates under the assumption of continued historical patterns. This projection implies that, under current patterns of disease management, healthcare utilization, and thyroid disease epidemiology, thyroid disease–related mortality among older adults may remain relatively stable with only modest year-to-year fluctuations over the near future.

#### Generalized additive model (GAM)

The GAM analysis revealed a significant nonlinear relationship between calendar year and AAMR. The smooth term for Year had an effective degrees of freedom (edf) of 4.201, with an F-statistic of 44.28 and *p* < 2 × 10^−16^, indicating that a simple linear term would be insufficient to adequately describe the complex temporal pattern. The GAM explained approximately 93.3% of the deviance, with an adjusted R^2^ of 0.916, demonstrating excellent goodness of fit compared to simpler parametric approaches. The fitted smoothed curve revealed a complex, slowly varying nonlinear pattern in AAMR from 1999 to 2020, with subtle but discernible indications of recent plateauing or mild increases in some years. Notably, this nonlinear pattern captures the recent trend reversals observed in age-stratified, regional, and racial/ethnic subgroup analyses (particularly evident in 65–74 year-olds, Hispanic populations, and Southern regions). The GAM-based forecasts for 2021–2030 project continuation of this nonlinear pattern into the future (Figure 7, green line), suggesting a more pronounced upward trajectory compared to the ARIMA baseline. The substantially wider 95% confidence intervals for GAM predictions reflect the inherent uncertainty in extrapolating nonlinear relationships beyond the observed data period.

#### Comparison of ARIMA and GAM approaches

The two forecasting models provide complementary insights into potential future trends (Figure 7). The ARIMA model, based on differenced, approximately stationary data, provides a conservative, nearly linear “baseline” projection that assumes continuation of historical average patterns without substantial structural change. This approach is advantageous for robust, transparent forecasting when future patterns are expected to resemble the past, and the narrow confidence intervals reflect confidence in the model’s assumptions. In contrast, the GAM, without imposing a predetermined functional form, captures subtle nonlinearities and temporal complexity inherent in the AAMR data and suggests the possibility of emerging upward or accelerating trends in thyroid disease–related mortality among older adults. The wider confidence intervals for GAM predictions acknowledge greater uncertainty when extrapolating nonlinear patterns, but this broader interval may more realistically capture plausible future scenarios given the recent pattern shifts observed across multiple demographic and geographic subgroups.

## Discussion

Using data from the CDC WONDER Multiple Cause of Death database, this study evaluated thyroid disease–attributed mortality among U.S. adults aged ≥65 years from 1999 to 2023. The overall AAMR declined from 4.690 to 3.482 per 100,000 population, corresponding to an AAPC of −1.22% and an EAPC of −1.43%. However, this decline was not uniform throughout the study period. Joinpoint analyses indicated that the most pronounced reductions occurred during the early-to-mid 2000s, whereas trends plateaued or reversed in certain age groups and geographic areas during 2015–2023. These findings suggest that the long-term reduction in thyroid disease–attributed mortality occurred within a changing epidemiological and healthcare context. Nevertheless, because the analysis was based on aggregated death certificate data, these temporal patterns should not be interpreted as evidence that specific changes in clinical management directly caused the observed decline.

Marked sex differences were observed in both the absolute mortality burden and temporal trends. Women accounted for 77.83% of all thyroid disease–attributed deaths and consistently had substantially higher AAMRs than men, with the female AAMR approximately 2.0–2.5 times the male rate. Although mortality declined in both sexes, men experienced a greater relative reduction over the full study period than women (−27.66% vs. −19.32%). Both sexes nevertheless showed evidence of trend deceleration or reversal after approximately 2010–2015. Thus, the overall reduction in mortality did not eliminate the substantial sex disparity in the absolute burden.

The higher AAMR among women is broadly consistent with the greater prevalence of thyroid disease, particularly autoimmune thyroid disorders, in women (34). Biological and immunological differences may contribute to this susceptibility, whereas greater healthcare utilization among women may increase the likelihood that thyroid disease is diagnosed and documented on death certificates. Older women also frequently have coexisting conditions such as osteoporosis, arrhythmias, and other chronic diseases that may complicate the clinical course of thyroid dysfunction (35). Conversely, men had lower absolute AAMRs but experienced a larger relative decline. Changes in the management of cardiovascular risk factors, including blood pressure, lipid, and glucose control, and advances in the recognition and acute management of severe hyperthyroidism and thyroid storm may have occurred alongside this trend (36–39). Differences in death certification practices by sex and clinical context may also have influenced the recorded rates. These explanations should be regarded as plausible contextual factors rather than mechanisms directly demonstrated by the present study.

Previous epidemiological research on thyroid disease has focused predominantly on prevalence and its associations with cardiovascular outcomes (6, 40), disorder of bone metabolism (41, 42), or cognitive impairment (43, 44). Nationwide studies examining long-term mortality trends specifically among deaths for which thyroid disease was recorded as the underlying cause remain relatively limited, particularly among adults aged ≥65 years. By analyzing 25 years of nationally representative data and focusing specifically on older adults, this study extends the available evidence on the population-level burden and temporal patterns of thyroid disease–attributed mortality.

The observed decline occurred during a period of substantial changes in the recognition, monitoring, and treatment of thyroid disease. These changes included broader use of thyroid function testing, continued monitoring of iodine nutrition, more standardized use of antithyroid medications and levothyroxine replacement therapy, and increased attention to thyroid function in perioperative and critical care settings (2, 45). Management of important comorbidities, including cardiovascular disease, metabolic disorders, and malignancies, also evolved during the study period (46–48). Such developments may have contributed to the broader clinical context in which thyroid disease–attributed deaths occurred. However, CDC WONDER does not contain individual-level information on diagnosis, treatment, disease severity, or treatment response. The present analysis therefore cannot determine whether these healthcare-related changes directly contributed to the observed mortality decline.

It is important to note that the COVID-19 pandemic may have temporarily altered mortality patterns in older adults and introduced disturbances into thyroid disease–related mortality rates (49, 50). To reduce the influence of the later pandemic period on model estimation, the forecasting models were fitted using data from 1999 to 2020, with 2021–2030 treated as the projection period. However, because the year 2020 was itself affected by the pandemic, this approach could not completely isolate pandemic-related effects. The direct and indirect effects of COVID-19 on thyroid function, healthcare utilization, death certification, and thyroid disease–attributed mortality require further investigation.

A methodological contribution of this study was the parallel application of ARIMA and GAM models to explore possible future mortality trajectories (51, 52). The ARIMA models was selected on the basis of favorable information criteria and satisfactory residual diagnostics. The absence of significant residual autocorrelation in the Ljung–Box test suggested that the model adequately represented the temporal dependence in the AAMR series from 1999 to 2020 (53). Its projections indicated a relatively stable, slowly declining trajectory through 2030, providing a conservative scenario based largely on continuation of the historical trend.

In contrast, the GAM used a smooth functions of calendar year to capture nonlinear changes in AAMR (54). The effective degrees of freedom (edf) for the smooth term exceeded 1, and the F-statistic was highly significant, indicating the presence of nonlinear components in the AAMR trend, such as changes in the slope of decline, plateau phases, or localized inflection points. The GAM achieved high goodness of fit (approximately 93.3% deviance explained, adjusted R^2^ = 0.916). Compared with ARIMA, the GAM suggested that the historical decline might attenuate or plateau during certain future periods. As medical technologies, clinical guidelines, population structure, and public health events evolve, long-term predictions are inherently uncertain. This is particularly true for flexible models such as GAM, which may be more sensitive to small changes in recent data when extrapolating far into the future. Therefore, we recommend interpreting the ARIMA model as a relatively robust baseline projection and the GAM as a more sensitive tool for detecting potential nonlinear or emerging trends. The two should be viewed as complementary, offering different but useful perspectives for public health decision-making, rather than as competing models where one is “right” and the other “wrong.”

Combining these results with existing pathophysiological and clinical evidence, several implications can be proposed: First, thyroid function assessment and follow-up should be further strengthened in older adults, particularly in high-risk groups such as those with established cardiovascular disease, diabetes, severe hepatic or renal impairment, or long-term polypharmacy, to avoid thyroid dysfunction being overlooked in the context of multiple comorbidities.

Second, from a sex-specific perspective, although women have higher AAMRs, the larger recent decline in men suggests that clinical practice should pay attention not only to the higher prevalence in women but also to potential underestimation or delayed diagnosis of thyroid dysfunction in older men.

Third, analysis of the spectrum of contributing causes (e.g., circulatory diseases, cancers, metabolic disorders) can help identify high-risk combinations of “thyroid disease + specific comorbidities” and provide targets for integrated care. For older patients with thyroid disease and concomitant cardiovascular disease or malignancy, more detailed evaluation and management of thyroid function may be warranted in routine clinical pathways.

Fourth, at the health policy level, combining ARIMA- and GAM-based forecasts can help anticipate the range of possible changes in thyroid disease–related mortality among older adults over the next decade. This can support more forward-looking planning in resource allocation for geriatric care, endocrinology and geriatric medicine services, and chronic disease management programs. If predictions indicate slower declines or potential rebounds in certain groups or regions, intensified interventions targeting thyroid disease and related comorbidities may be needed in those priority populations and areas.

This study has several strengths. It used a nationally representative mortality database spanning 25 years and applied a clearly defined outcome restricted to deaths in which thyroid disease was recorded as the underlying cause. The use of both crude and age-adjusted mortality rates, together with Joinpoint regression and complementary trend measures, enabled characterization of overall and segment-specific temporal patterns. Stratification by demographic, geographic, and urban–rural characteristics provided a broad assessment of population differences. The analysis of contributing causes offered additional information regarding conditions recorded alongside thyroid disease, whereas the combined use of ARIMA and GAM provided complementary exploratory perspectives on possible future trends.

However, several limitations should be considered. First, this study relied on death certificate data and included only deaths for which thyroid disease was recorded as the underlying cause. Misclassification and underreporting are possible, particularly among older adults with multiple comorbidities. Therefore, the findings reflect death certificate–based thyroid disease–attributed mortality rather than the total mortality burden involving thyroid disease.

Second, CDC WONDER provides aggregated data; thus, this study was descriptive and ecological, and the observed temporal and subgroup differences should not be interpreted causally. Detailed clinical and individual-level information, including disease severity, treatment, thyroid function, socioeconomic status, healthcare access, and comorbidity severity, was unavailable, limiting control of potential confounding.

Third, ICD-10 codes E00–E07 include clinically heterogeneous non-malignant thyroid disorders with different courses and prognoses. They were analyzed collectively to characterize the overall population-level burden and to support stable subgroup, geographic, and forecasting analyses. However, this approach may have obscured subtype-specific patterns, which should be examined in future studies.

Fourth, state-level estimates may be unstable in states or years with relatively few deaths; therefore, some interstate variation may reflect statistical fluctuation rather than true differences in mortality burden.

Finally, the influence of COVID-19 could not be fully isolated. In addition, the ARIMA and GAM projections were based on a limited number of annual observations and should be regarded as exploratory model-based scenarios rather than precise predictions of future mortality.

## Conclusion

From 1999 to 2023, the age-adjusted mortality rate among U.S. adults aged ≥65 years with thyroid disease recorded as the underlying cause of death showed an overall decline, although the mortality burden remained notable and differed across population groups. Women consistently had higher absolute mortality rates than men, whereas men showed a steeper recent decline, indicating persistent sex differences in mortality patterns that may reflect differences in thyroid disease prevalence, comorbidity profiles, healthcare use, or death certificate coding practices. ARIMA and GAM models produced different projected trajectories, highlighting uncertainty in the future burden of thyroid disease–attributed mortality among older adults. These findings should be interpreted as descriptive epidemiological patterns rather than evidence of causal effects. Continued surveillance and further studies using individual-level clinical data are warranted to better characterize high-risk populations and inform future public health planning.

## Data Availability

The original contributions presented in the study are included in the article/Supplementary material, further inquiries can be directed to the corresponding author.
